# Draft Genome of the Nitrogen-Fixing Bacterium *Pseudomonas stutzeri* Strain KOS6 Isolated from Industrial Hydrocarbon Sludge

**DOI:** 10.1128/genomeA.00072-12

**Published:** 2013-01-31

**Authors:** Tatiana V. Grigoryeva, Aleksandr V. Laikov, Rimma P. Naumova, Aleksandr I. Manolov, Andrey K. Larin, Irina Y. Karpova, Tatiana A. Semashko, Dmitry G. Alexeev, Elena S. Kostryukova, Rudolf Müller, Vadim M. Govorun

**Affiliations:** a Institute of Fundamental Medicine and Biology, Kazan (Volga Region) Federal University, Kazan, Russia; b Scientific Research Institute of Physical-Chemical Medicine, Moscow, Russia; c Moscow Institute of Physics and Technology, Moscow, Russia; d Institute of Technical Biocatalysis, Technical University of Hamburg-Harburg, Hamburg, Germany

## Abstract

Here we present a draft genome of *Pseudomonas stutzeri* strain KOS6. This strain was isolated from industrial hydrocarbon sludge as a diazotrophic microorganism. It represents one of the major parts of the culturable community of the waste and has potential importance for phytoremediation technology.

## GENOME ANNOUNCEMENT

*Pseudomonas stutzeri* is a Gram-negative bacterium widely distributed in the environment, with extremely broad phenotypic and genotypic diversity. It can rarely be found as an opportunistic pathogen in humans (1). These bacteria are known to be nutritionally versatile and especially interesting because of specific metabolic abilities (denitrification, degradation of aromatic compounds, nitrogen fixation), which were found in some of the strains (2).

Genome assemblies for ten strains of *P. stutzeri*, including one type strain (clinic CGMCC 1.1803 [3]), one naphthalene-degrading strain (CCUG 29243 [4]), two nitrogen-fixing strains (A1501 [5], DSM 4166 [6]), one carbazole-degrading (XLDN-R [7]), one arsenite-oxidizing strain (TS44 [8]), some model strains (denitrification CCUG 16156 [9], T13 [10]), a natural transformation strain (DSM 10701 [11]), and a lactate utilization strain (SDM-LAC [12]), are already available at GenBank. We used them for comparison with the genome of strain KOS6.

*P. stutzeri* strain KOS6 was isolated from industrial hydrocarbon sludge of a chemical enterprise in Kazan, Russian Federation. This strain was capable of biological nitrogen fixation of about 23 nmol C_2_H_4_ ml^-1^ h^-1^ measured by Hardy’s acetylene method and has additional useful properties—like other diazotrophic isolates from hydrocarbon sludges (13).

The genome of strain KOS6 was sequenced using the PGM sequencer system (Life Technologies). For the assembly we did four sequencing runs and got two fragmented and two mate-pair libraries with 483-fold overall coverage. Assemblies were done using Mira 3.4.0 and CLC Genomics 5.1, with manual postprocessing with Vector NTI software to fill the gaps. The obtained genome sequence included 79 contigs (>200 bp in size), with a calculated genome size of 5,014,616 bp with a G+C mol% of 62.55%. The genome was annotated using the NCBI Prokaryotic Genome Annotation Pipeline (PGAAP) (http://www.ncbi.nlm.nih.gov/genomes/static/Pipeline.html).

Multiple-genome comparisons were performed with BLAST. Strain KOS6 was most closely related to strain CCUG 29243 (blast total score, 4.560e+06, 70% query coverage), followed by strain DSM 4166 (4.270e+06, 68%), strain ATCC 17588 (4.272e+06, 67%), and strain A1501 (4.127e+06, 66%).

Along with housekeeping genes and a typical denitrification system, the genome of KOS6 contains a number of genes which are important for phytoremediation technology. These genes are involved in plant growth promotion, in resistance to toxicants, and in aromatic and polycyclic aromatic hydrocarbon degradation. In contrast to genomes of nitrogen-fixing *P. stutzeri* (A1501, DSM 4166), there are genes for type VI secretion systems, for transport systems, and for additional pathways of naphthalene degradation in our strain. Moreover, strain KOS6 contains 37 unknown and 38 annotated genes with homology to *Tolumonas auensis* DSM 9187, which are presumably related to survival under extreme conditions in the waste.

Future investigations of the relationships between the newly discovered genes will help to further our understanding of the evolution and adaptation of *P. stutzeri* as well as broaden our knowledge of the ecology and physiology of nitrogen-fixing bacteria.

### Nucleotide sequence accession numbers. 

This Whole Genome Shotgun project has been deposited at DDBJ/EMBL/GenBank under the accession number AMCZ00000000. The version described in this paper is the first version, AMCZ01000000.
